# A Comparative Study on Suitability of Model-Free and Model-Fitting Kinetic Methods to Non-Isothermal Degradation of Lignocellulosic Materials

**DOI:** 10.3390/polym13152504

**Published:** 2021-07-29

**Authors:** Hamayoun Mahmood, Ahmad Shakeel, Ammar Abdullah, Muhammad Ilyas Khan, Muhammad Moniruzzaman

**Affiliations:** 1 Department of Chemical, Polymer & Composite Materials Engineering, University of Engineering & Technology, New Campus, Lahore 54890, Pakistan; engr.hamayoun@uet.edu.pk; 2 Department of Hydraulic Engineering, Faculty of Civil Engineering and Geosciences, Delft University of Technology, Stevinweg 1, 2628 CN Delft, The Netherlands; 3 Department of Chemical Engineering, University of Engineering & Technology, Lahore 54890, Pakistan; ammarmayo1@gmail.com; 4 Department of Chemical Engineering, King Khalid University, Abha 62529, Saudi Arabia; mikhan24@yahoo.co.uk; 5 Department of Chemical Engineering, Universiti Teknologi PETRONAS, Bandar Seri Iskandar, Perak 32610, Malaysia; m.moniruzzaman@utp.edu.my

**Keywords:** lignocellulosic fuels, kinetic modeling, thermogravimetry, isoconversional modeling, model-fitting methods

## Abstract

The thermal kinetic modeling is crucial for development of sustainable processes where lignocellulosic fuels are a part of chemical system and their thermal degradation eventuates. In this paper, thermal decomposition of three lignocellulosic materials (bagasse, rice husk, and wheat straw) was obtained by the thermogravimetric (TG) technique and kinetics was analyzed by both model-fitting and isoconversional (model-free) methods to compare their effectiveness. Two models selected from each class include Arrhenius and Coats–Redfern (model-fitting), and Kissinger–Akahira–Sunose (KAS) and Flynn–Wall–Ozawa (FWO) (model-free). The formal model-fitting approach simulating the thermal decomposition of solids by assuming a fixed mechanism was found to be unduly facile. However, activation energy (E) values calculated from two model-fitting techniques were considerably different from each other with a percentage difference in the range of 1.36% to 7.65%. Particularly, both model-fitting methods predicted different reaction mechanism for thermal disintegration of lignocellulosic materials (two-dimensional diffusion (D2) by Arrhenius and one-dimensional diffusion (D1) by Coat–Redfern method). Conversely, the model-free routine offers a transformation of mechanism and activation energy values throughout reaction and is, therefore, more authentic to illustrate the complexity of thermal disintegration of lignocellulosic particles. Based on the model-free kinetic analysis, the lignocellulosic materials may be devised in following order of activation energy: rice husk > bagasse > wheat straw, by both KAS and FWO methods with a percentage difference no more than 0.84% for fractional conversion up to 0.7. Isoconversional approach could be recommended as more realistic and precise for modeling non-isothermal kinetics of lignocellulosic residues compared to model-fitting approach.

## 1. Introduction

Access to energy is crucial to subdue poverty, boost economic expansion and employment opportunities and uphold the provision of social services, such as healthcare and education for sustainable human development. Energy access and energy poverty have been increasingly realizing as essential issues for society as an integral part of global development agendas. It is substantial to cope with these issues in the framework of two other considerable socio-ecological challenges of present time, that is, energy certainty and environmental sustainability. This threefold issue is imputed as the “energy trilemma” by the World Energy Council (WEC) [1,2]. Lignocellulosic biomass, as an ideal renewable energy source, offers numerous advantages including renewability, abundant reserves, cheap, and carbon neutrality. In contrast to other renewable energy resources, it can be utilized for generation of heat, power, and transportation fuels, and is proficient to generate continuous energy under wide range of scales. The lignocellulosic feedstocks resources are more evenly distributed geographically compared to fossil sources, conforming the security of supply to a large extent [3,4]

However, lignocellulosic biomass-based fuels constitute only about 10% of annual energy consumption worldwide [5]. The large-scale processing of lignocellulosic materials into fuel and valuable products through thermochemical conversion, and numerous heterogeneous reactions with indefinite kinetics entailed at different phases of thermal degradation is one of the critical problems. Particularly, the intricate 3D structure of lignocellulose matrix is extremely cumbersome for engineers to design reactors for biomass thermochemical transformation processes [6]. The thermal decomposition kinetics of lignocellulosic materials can be computed by simulation of the rates of thermal degradation data by a suitable kinetic model. Accurate computation of kinetic parameters for lignocellulose thermal decomposition is indispensable to forecast real-time material performance and lifetime estimation, modeling the combustors and gasifiers and to optimize the operating parameters [7,8]. For the fruitful utilization of computational fluid dynamics (CFD) simulation to lignocellulose thermochemical conversion, it is necessary to predict kinetic parameters of devolatilization process [9].

Thermogravimetry is a frequently employed analytical method to measure the thermal degradation of solid materials and subsequently formulate kinetic models to illustrate the pyrolysis process quantitatively [10,11,12]. The challenge in formulating the kinetic expressions is mainly due to two reasons: (i) pyrolysis of lignocellulosic materials entails manifold parallel and series disintegration stages occurring concurrently and (ii) thermal disintegration of lignocellulosic material is extremely heterogeneous. These attributes always stimulate the scientists to formulate more rigorous kinetic models to describe solid fuel degradation. Two types of models have been developed in this regard viz. model-based and isoconversional or model-free techniques [13]. Model-fitting was the first and exceedingly famous approach used to model solid-state kinetics. In this method, first a reaction mechanism is presumed and applied to TGA data. The most suitable reaction model is then picked on the basis of quality of the regression fit [14]. Whereas, no reaction model is presumed in model-free methods and kinetic information is deduced from mathematical relations formulated and kinetic parameters are computed without supposing a reaction mechanism [15,16]. 

The quest for appropriate category of models that can precisely depict the kinetics of thermal degradation of lignocellulosic fuels necessitates a descriptive comparison among different categories of models. Numerous examples of thermal degradation kinetics of different solid materials either by isoconversional or by model-fitting methods can be found in literature [17,18,19]. A few studies provided a comparison of model-free and model-fitting approaches for different solid fuels [9,20] but an elaborated comparison of both categories for efficient modeling of thermal degradation behavior of lignocellulosic materials is lacking. 

Here, kinetics for thermal disintegration of three lignocellulosic fuels (wheat straw, rich husk, and bagasse) has been evaluated and compared by following both model-fitting and isoconversional methods to inspect reliability of each category of models to describe the pyrolysis kinetics of lignocellulosic biomass.

## 2. Materials and Methods 

The biomass samples (wheat straw, rich husk, and bagasse) were collected locally from Lahore, Pakistan and thermogravimetric analysis was conducted on SDT Q600, TA Instrument, New Castle, DE, USA. The samples with a particle size of <250 µm were heated from 25 °C to 600 °C at different heating rates of 10, 20, and 30 °C/min. The heat transfer barrier could be evaded by employing smaller sized particles under low heating rate [21]. High-purity nitrogen gas was continuously flushed through the instrument at a flow rate of about 100 mL/min to maintain inert atmosphere. The TGA was performed using a small amount of sample (0.5 mg) to avoid exothermic self-heating and thermal gradients.

## 3. Kinetic Modelling

The thermal deterioration of biomass is a tortuous process and transport phenomena has not precisely been described. Many authors have represented the rate of thermal decomposition by following expression [22]:

(1) $$\frac{d\alpha }{dt}=k\left(T\right)\times f\left(\alpha \right)$$

where rate constant k varies with the absolute temperature (T) following Arrhenius equation, $k\left(T\right)=Ae^{-E/RT}$, in which R represents the universal gas constant (R = 8.314 J/mol K), A stands for frequency factor, and E is activation energy [23]. Thus:

(2) $$\frac{d\alpha }{dt}=Ae^{-\frac{E}{RT}}\;f\left(\alpha \right)$$

where α represents the fractional conversion and is given as:

(3) $$\alpha =\frac{m_{i}-m_{t}}{m_{i}-m_{f}}$$

where $m_{i}$, $m_{t}$ and $m_{f}$ are the initial, instantaneous and final mass of biomass sample at any temperature T during TGA analysis. Under non-isothermal conditions, heating rate (β) changes with time to give the following relation:

(4) $$\frac{d\alpha }{dT}=\frac{d\alpha }{dt}\times \frac{1}{\beta }$$

And, thus, Equation (2) becomes,

(5) $$\frac{d\alpha }{dT}=\frac{A}{\beta }e^{-\frac{E}{RT}}\times f\left(\alpha \right)$$

Different forms of f(α) in conformity with different reaction mechanisms have been summarized in Table 1 [24].

### 3.1. Model-Fitting Approach

#### 3.1.1. Arrhenius Model

Many researchers have applied this model to compute kinetic parameters of solid materials [23,24]. In this model-fitting approach, f(α) can be replaced by the relation $f\left(\alpha \right)={\left(1-\alpha \right)}^{n}$, in which n is reaction order, Equation (5) becomes,

(6) $$\frac{d\alpha }{dT}=\frac{A}{\beta }e^{-\frac{E}{RT}}\times {\left(1-\alpha \right)}^{n}$$

For a specific reaction mechanism, a plot of ln[(dα/dT)/f(α)] against 1/T should provide a straight line and E values can be estimated from the slope (−E/R) of that line. The linearity (R^2^, correlation coefficient) value could be an indication for satisfactoriness of each function model on reaction.

#### 3.1.2. Coats-Redfern Model

In this model, temperature integral is determined by using a asymptotic series expansion. The following form of Equation (6) has been proposed by many researchers to apply Coats–Redfern model for solid state kinetics of different lignocellulosic materials [9,10]:

(7) $$ln\left(\frac{g\left(\alpha \right)}{T^{2}}\right)=ln\frac{AR}{\beta E}\left(1-2\frac{RT}{E}\right)-\frac{E}{RT}$$

in which g(α) implicates the mechanism function for a particular reaction model during thermal disintegration of material, as sum up in Table 1. By comparing Equation (7) with the expression y=mx+c. The satisfactory of each function mechanism can be assessed through inspecting the correlation coefficient, R^2^ which indicates the linearity of ln[g(a)/$T^{2}$] versus 1/T plot and activation energy can be determined from the slope.

### 3.2. Model-Free Approach

#### 3.2.1. Flynn–Wall–Ozawa (FWO) Method

Various integral isoconversional methods have also implied possible solution of Equation (6) that differ in predictions of the temperature integral. The following linear equation may be represented as the general model for most of these approximations [25]:

(8) $$ln\beta_{i}/T_{\alpha ,i}^{B}=Const-C\left(E_{\alpha }/RT_{\alpha }\right)$$

where the values of parameters B and C rely on the evaluation of temperature integral. The FWO model has been considered as the exceedingly reliable model for computation of thermal decomposition kinetics and is given by the expression as follows [26]:

(9) lng(α)=logAE/R−lnβ−2.315−0.4567E/RT

TGA data of solid material under various heating rates can be modeled using Equation (9) and the activation energy (E) can be computed from the slope of ln(β) vs. 1/T graph for specific conversion α. 

#### 3.2.2. Kissinger–Akahira–Sunose (KAS) Method 

The KAS method, a model-free technique, has been extensively applied for thermal kinetics of solid fuel materials and can be primitively attained from the derivative of Equation (5) as shown below:

(10) ∫0∞dα f(α)=Aβ ∫0Te−ERTdT

Let u=E/RT, then above expression becomes:

(11) $$\int_{0}^{\infty }\frac{d\alpha }{\;f\left(\alpha \right)}=\frac{AE}{\beta R}\;\int_{x}^{\infty }u^{-2}e^{-u}du=\;\frac{AE}{\beta R}\;P\left(x\right)$$

By setting $g\left(\alpha \right)=\int_{0}^{\infty }\frac{d\alpha }{\;f\left(\alpha \right)}$, Equation (11) can be written as:

(12) $$g\left(\alpha \right)=\frac{AE}{\beta R}P\left(x\right)$$

Numerous approximations of P(x) can be used depending upon the particular method. The KAS approach apply the empirical correlation: $P\left(x\right)=x^{-2}e^{-x}\;$[21,27], which transforms Equation (12) into:

(13) $$ln\left(\frac{\beta }{T^{2}}\right)=ln\left(\frac{AE}{R\times g\left(x\right)}\right)-\;\frac{E}{R}\cdot \frac{1}{T}$$

**Figure 1:**
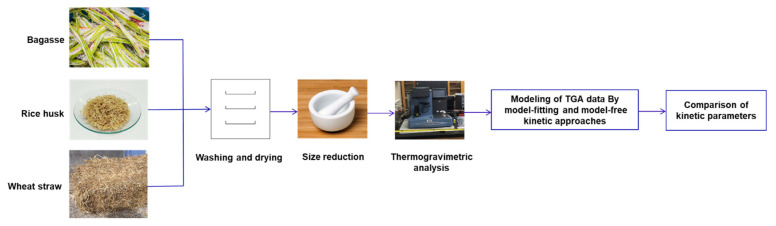
Schematic representation for comparison of thermal kinetic modeling by model-fitting and model-free methods of different lignocellulosic materials.

In this method, the knowledge of the conversion-dependent functions (g(α) or f(α)) is not required and it only presumes that the thermal disintegration of material follows the same mechanism of reaction for a given conversion. For a constant α, a plot of $ln\left(\beta /T^{2}\right)$ versus 1/T should render a straight line and E can be obtained from the slope. Figure 1 portrays the schematic representation for comparison of thermal kinetic modeling by model-fitting and model-free methods as performed in the current study.

## 4. Results and Discussion

### 4.1. Thermal Decomposition Behavior of Lignocellulosic Fuels

**Figure 2:**
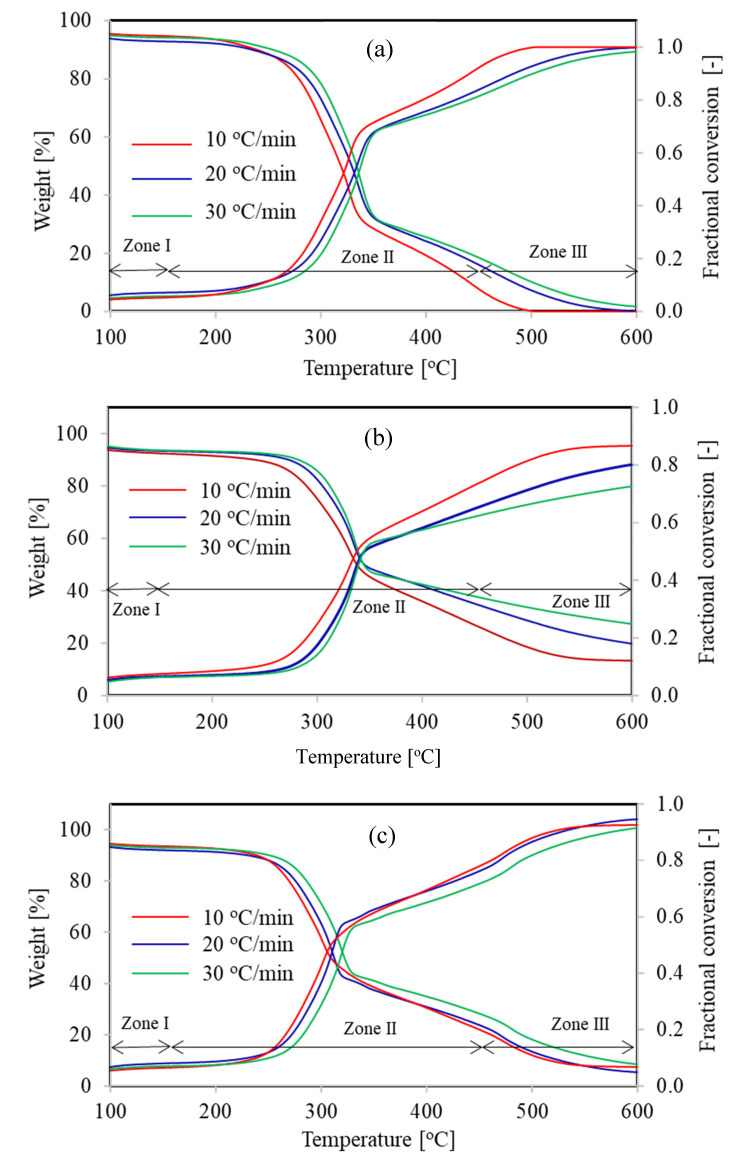
Thermal degradation profiles of (**a**) Bagasse, (**b**) Rice husk and (**c**) Wheat straw under different heating rates.

Generally, thermal disintegration of lignocellulosic materials is completed in three steps, viz. water devolatilization, hemicellulose/cellulose decomposition, and lignin decomposition. The decomposition phenomenon of any biomass can be considered as the superposition of these principal constituents [28,29]. The weight loss and conversion as a function of temperature for wheat straw, bagasse, and rice husk under three different heating rates are displayed in Figure 2, whereas Table 2 provides chemical composition of lignocellulosic samples. Three different zones can be identified depending upon the phenomenon occurring during the thermal degradation of biomass (see Figure 2). For instance, moisture evaporation takes place in zone I, whereas in zone II mainly decomposition of hemicellulose-cellulose occurs and degradation of lignin and inorganics in fuel accompanies in zone III. Due to its complex structure thermal degradation of lignin overlaps with that of hemicellulose and cellulose. Thermal degradation zones of hemicellulose and cellulose coincide with each other so that two different endotherms cannot be distinguished for the cellulose and hemicellulose degradation in the DTG curve (not shown). Maximum weight loss of the material occurs in zone II as decomposition of major components (cellulose and hemicellulose) takes place in this region and pyrolysis rate rises rapidly [30]. 

Figure 2 also describes the impact of heating rate on the thermal decomposition of lignocellulosic materials. It could be noted from Figure 2 that an increase in heating rate provoked the reaction field towards a higher temperature region. This behavior is due to different residence time of fuel particles in the reactor under different heating rates. Heating rates and residence time are inversely commensurate to each other, when heating rate will be low enough the residence time will be high and thermal gradients sneak into the inner core of the solid fuel particles. Although under high heating rate operations, thermal gradients even not spread uniformly into the particle due to low residence time resulting in less assertive peaks in DTG curve [31]. This might be the primary cause for confined temperature range at low heating rates and broader temperature range at high heating rates, as shown in Table 3. It is also evident from Figure 2 that reactivity of lignocellulosic materials increases with enhancement in heating rate.

### 4.2. Comparative Kinetic Analysis of Lignocellulosic Fuels

Pyrolysis kinetics of bagasse, rice husk, and wheat straw was evaluated by following two isoconversional models and two model-fitting methods. Different reaction mechanisms for both model-fitting methods were used, as listed in Table 1, to assess the best promising reaction model having well conformity with the experimental data. Table 4 depicts the estimated activation energy (E) values from Coats–Redfern and Arrhenius models along with the corresponding values of correlation coefficient, R^2^. Both models show the analogous trend of E values for all three lignocellulosic materials. The highest E values of 92.73 kJ/mol and 89.41 kJ/mol were observed for bagasse under Arrhenius and Coats–Redfern models, respectively. Although rice husk exhibits lowest values of activation energy viz. 77.18 kJ/mol (Arrhenius model) and 71.49 kJ/mol (Coats–Redfern model). The different E values may be attributed to different composition of biomass particles as each lignocellulosic constituent possess significantly different value of activation energy [32]. The highest value of E for bagasse may be due to its higher volatile and lower ash contents in contrast to rice husk as shown in Table 2 [33,34]. If we compare the results of both model-fitting techniques, fairly dissimilar values of activation energy and R^2^ could be noted. On the other hand, Arrhenius model predicted that thermal disintegration of all three lignocellulosic materials followed D2 (two-dimensional diffusion) mechanism while Coats–Redfern model appraised that D1 (one-dimensional diffusion) could be applicable for all fuel particles. Model-fitting methods did not furnish corresponding results with each other, as also evident from low values of R^2^.

**Figure 3:**
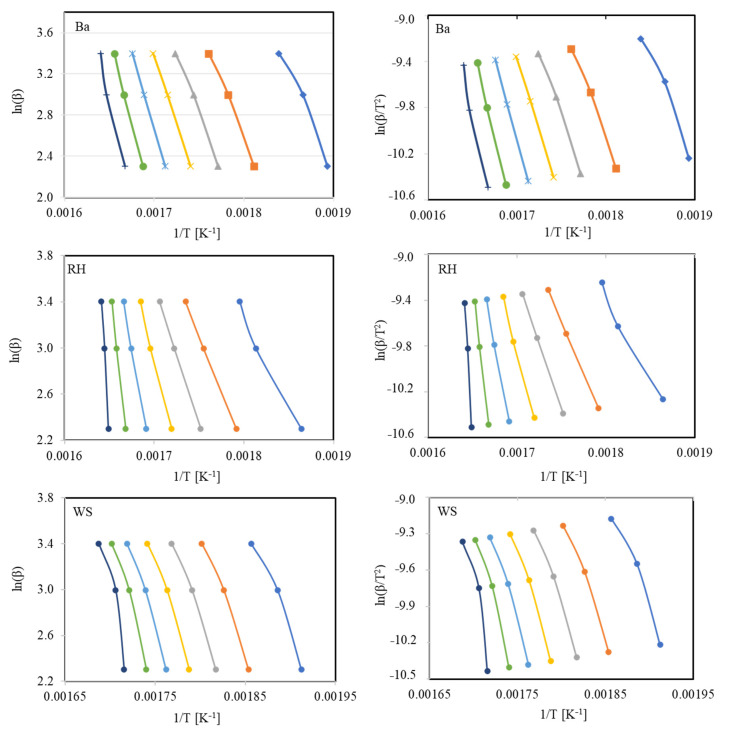
Graphical depiction of isoconversional models for bagasse (Ba), rice husk (RH), and wheat straw (WS).

In case of FWO integral isoconversional model, a graph of ln(β) vs. 1/T for specific conversion renders a straight line with slope −E/R, while following KAS approach a straight line plot of $ln\left(\beta /T^{2}\right)$ against 1/T was obtained with slope −E/R (Figure 3). The values of E and correlation coefficient R^2^ calculated thereof are presented in Table 5. It could be noted from Table 5 that the activation energy values determined from both model-free methods exhibited trivial difference. Particularly, excellent capability of model-free isoconversional models to better fit the experimental thermal degradation profiles of lignocellulosic materials is vivid from R^2^ values, which are near to unity. Similar output from model-free approach for different solid fuels kinetics has been reported in the literature as well [10,35,36]. 

**Figure 4:**
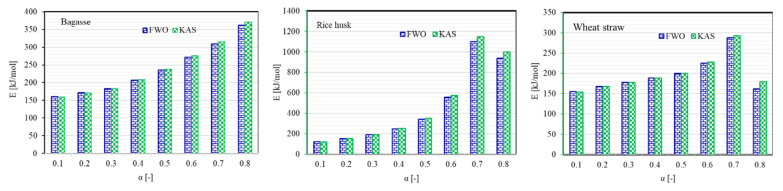
Activation energy as a function of fractional conversion for different lignocellulosic fuels using two model-free techniques.

**Figure 5:**
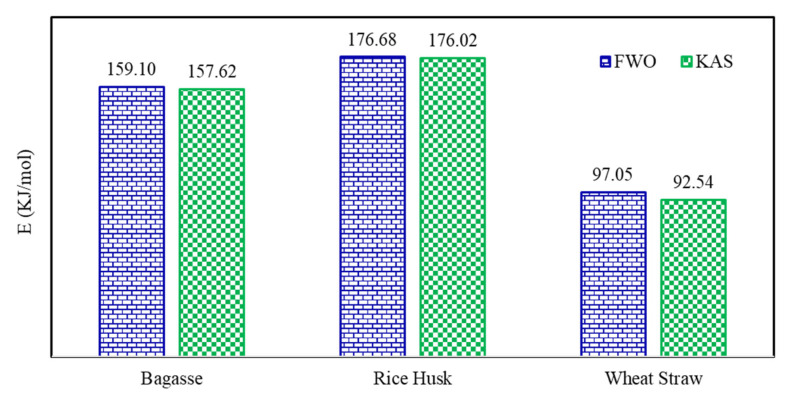
Comparison of average values of activation energy (E) of lignocellulosic materials by isoconversional methods.

From the result of two model-free methods, lignocellulosic materials could be devised in succeeding order of activation energy: rice husk > bagasse > wheat straw. The mutation in activation energy with conversion for different lignocellulosic fuels has also been drawn in Figure 4. A substantial variation in the E values with the progress of thermal disintegration of material is evident from Figure 4 and the variation is even extensive at higher conversion. Thus, from a kinetic standpoint, it may be proposed that thermal decomposition mechanism of lignocellulosic matrices is an intricate phenomenon which may include unzipping de-polymerization, cis-elimination, intra and intermolecular trans-esterification, radical or hydrolytic degradation, random scission, etc. Hence, thermal degradation process should not be represented by a unique set of kinetic parameters for the entire conversion span [37]. The E values estimated from both isoconversional models were very close to each other for α < 0.6, and for fractional conversion larger than 0.6 the percentage difference between E values becomes appreciable. Nevertheless, the likelihood of intricate thermal degradation process does not deny the use of model-free approach, as this approach offer kinetic parameters by employing multiple single step equations for a specific temperature span associated with each fractional conversion [38,39]. A comparison of average E values for the whole conversion range from both FWO and KAS models is presented in Figure 5.

It could be suggested from the results shown in Table 4 and Table 5 that model-free techniques are more veritable and authentic in comparison with model-fitting approach for simulation of thermal deterioration of lignocellulosic particles. The employment of the model-fitting methods to predict kinetics by simulating TGA usually unable to incorporate temperature dependence of the reaction model, f(α). Consequently, virtually any f(α) can adequately fit the experimental data at the expense of extreme deviations in the kinetic parameters, which offsets the difference between the assumed form of f(α) and the true but unexplored reaction model. In the literature, this compensation effect has not been clarified completely but it was generally ascribed to the thermal lag [40,41], physicochemical, or mathematical causes [42]. Therefore, employment of the model-fitting methods under single heating-rate data have been reported to afford highly uncertain kinetic parameters values [20,43,44]. Low values of R^2^ were obtained for simulation of TGA data by both model-fitting methods and E values calculated form each model were also considerably different from each other (Table 5). It was mentioned by Holstein et al. (2005) that a particular set of experimental measurement could be fitted steadily well by numerous pairs of kinetic parameters in model-fitting approach [45].

Conversely, both model-free methods produced fairly similar activation energy values for an almost entire investigated conversion range. The relatively acute values of R^2^ offer their immense ability to simulate the experimental TGA data. Isoconversional models furnish to estimate alteration of activation energy with α, which indicate their capability to undertake the occurrence of numerous homogeneous as well as heterogeneous pathways during thermal decomposition phenomenon. Therefore, isoconversional models may be recommended for more reliable and precise prediction of thermal disintegration kinetics of lignocellulosic fuels.

## 5. Conclusions

A comparative suitability of isoconversional and model-fitting kinetic approaches to model experimental thermal disintegration behavior of different lignocellulosic materials including bagasse, rice husk and wheat straw was studied. Two models from each class of approach were chosen entailed Flynn–Wall–Ozawa (FWO) and Kissinger–Akahira–Sunose (KAS) (model-free or isoconversional), and Arrhenius and Coat–Redfern (model-fitting). Both model-fitting methods afforded considerably dissimilar values of activation energy with percentage difference in the range of 1.36% to 7.65% and predicted different reaction mechanisms for bagasse, rice husk and wheat straw. Two-dimensional diffusion (D2) mechanism was predicted by Arrhenius model while Coats–Redfern model suggested one dimensional diffusion (D1) for thermal degradation of all lignocellulosic materials. Conversely, both isoconversional methods predicted the E values as a function of fractional conversion (α) within the extent of 0 ≤ α ≥ 0.8. The values of correlation coefficient R^2^ were nearly unity for both isoconversional methods in contrast to model-fitting methods with average values of E as 159.1, 176.68, and 97.05 kJ/mol by FWO and 157.62, 176.02, and 92.54 kJ/mol by KAS method for bagasse, rice husk, and wheat straw, respectively. Model-free isoconversional techniques may be recommended as more realistic and accurate to model non-isothermal pyrolytic kinetics of lignocellulosic materials.

## Figures and Tables

**Table 1:** Various reaction mechanism for Arrhenius and Coats–Redfern model [24].

Mechanism	f(α)	g(α)	Abbreviation
Power law	$2\alpha^{1/2}$	$\alpha^{1/2}$	P2
Power law	$3\alpha^{2/3}$	$\alpha^{1/3}$	P3
Power law	$4\alpha^{3/4}$	$\alpha^{1/4}$	P4
Avarami-Eroféve	$2\left(1-\alpha \right){\left[-\ln\left(1-\alpha \right)\right]}^{1/2}$	${\left[-\ln(1-\alpha )\right]}^{1/2}$	A2
Avarami-Eroféve	$3\left(1-\alpha \right){\left[-\ln\left(1-\alpha \right)\right]}^{2/3}$	${\left[-\ln(1-\alpha )\right]}^{1/3}$	A3
Avarami-Eroféve	$4\left(1-\alpha \right){\left[-\ln\left(1-\alpha \right)\right]}^{3/4}$	${\left[-\ln(1-\alpha )\right]}^{1/4}$	A4
Contracting Sphere	$2{\left(1-\alpha \right)}^{1/2}$	$[1-\left(1-\alpha {)}^{1/2}\right]$	R2
Contracting Cylinder	$3{\left(1-\alpha \right)}^{2/3}$	$[1-\left(1-\alpha {)}^{1/3}\right]$	R3
One Dimensional Diffusion	1/2α	$\alpha^{2}$	D1
Two Dimensional Diffusion	${\left[-\ln\left(1-\alpha \right)\right]}^{-1}$	[(1−α)·ln(1−α)]+α	D2
Three Dimensional Diffusion–Jander	$\frac{3{\left(1-\alpha \right)}^{\frac{2}{3}}}{\left[2\left(1-{\left(1-\alpha \right)}^{\frac{1}{3}}\right)\right]\;}$	${\left[1-{\left(1-\alpha \right)}^{1/3}\right]}^{2}$	D3
Three-Dimensional Diffusion-GB	$3/2\left(1-\alpha^{\frac{-1}{3}}-1\right)$	$1-\frac{2\alpha }{3}-{\left(1-\alpha \right)}^{2/3}$	D4
First Order	(1−α)	−ln(1−α)	F1
Second Order	${\left(1-\alpha \right)}^{2}$	${\left(1-\alpha \right)}^{-1}-1$	F2
Third Order	${\left(1-\alpha \right)}^{3}$	$[\left(1-\alpha {)}^{-2}-1\right]/2$	F3

**Table 2:** Proximate and carbon-sulphur analysis of lignocellulosic samples.

		Bagasse	Rice Husk	Wheat Straw
Proximate Analysis (%)	MC	7.00	7.94	6.40
	VM	75.00	56.19	71.34
	FC	10.00	11.75	9.67
	Ash	8.00	24.13	12.59
C-S Analysis (%)	C	43.55	34.35	43.50
	S	0	0.28	0

MC = moisture content, FC = fixed carbon, VM = volatile matter, C = carbon, S = sulphur.

**Table 3:** Temperature ranges estimated for different zones of thermal degradation profiles performed at three different heating rates.

Heating Rate	10 °C/min			20 °C/min			30 °C/min		
Zone	I	II	III	I	II	III	I	II	III
Bagasse	40–114	114–362	362–600	40–123	123–376	376–600	40–130	130–385	385–600
Rice Husk	40–119	119–365	365–560	40–128	128–374	374–568	40–147	147–387	387–576
Wheat Straw	40–117	117–355	355–561	40–127	127–365	365–589	40–140	140–374	374–698

**Table 4:** Comparison of activation energies (E) predicted from model-fitting methods for all three lignocellulosic materials.

Biomass	Arrhenius Model			Coats Redfern Model			% Difference
	Mechanism	E (kJ/mol)	R^2^	Mechanism	E (kJ/mol)	R^2^	
Bagasse	D2	92.73	0.92	D1	89.41	0.96	3.65
Rice husk	D2	77.18	0.84	D1	71.49	0.89	7.65
Wheat straw	D2	86.22	0.89	D1	85.05	0.94	1.36

**Table 5:** Comparison of E Values predicted form model-free FWO and KAS methods for all three lignocellulosic materials.

Biomass	α	FWO Model		KAS Model		% Difference *
		E (kJ/mol)	R^2^	E (kJ/mol)	R^2^	
Bagasse	0.10	160.21	0.98	159.63	0.98	0.36
	0.20	171.81	0.99	171.43	0.99	0.22
	0.30	183.48	1.00	183.50	0.99	0.01
	0.40	206.82	1.00	207.91	1.00	0.53
	0.50	234.95	1.00	237.35	1.00	1.02
	0.60	271.66	1.00	275.84	1.00	1.53
	0.70	309.07	0.98	315.10	0.98	1.93
	0.80	362.33	0.96	371.02	0.96	2.37
Rice husk	0.10	122.42	0.99	119.70	0.98	2.25
	0.20	153.62	1.00	152.19	1.00	0.94
	0.30	190.22	1.00	190.50	1.00	0.15
	0.40	247.91	1.00	251.04	1.00	1.25
	0.50	342.65	1.00	350.57	1.00	2.28
	0.60	555.63	1.00	574.51	1.00	3.34
	0.70	1100.40	0.99	1147.51	0.99	4.19
	0.80	938.95	0.72	998.00	0.73	6.10
Wheat straw	0.10	155.04	0.97	154.28	0.96	0.49
	0.20	168.28	0.99	167.93	0.98	0.21
	0.30	177.89	0.99	177.87	0.99	0.01
	0.40	188.17	0.99	188.54	0.99	0.20
	0.50	199.99	0.99	200.84	0.98	0.42
	0.60	225.45	0.98	227.51	0.97	0.91
	0.70	287.77	0.89	292.95	0.88	1.78
	0.80	−161.73	0.22	−180.06	0.24	10.73

* Calculated based on the formula (y_1_ − y_2_)/(y_1_ + y_2_/2) × 100.

## Data Availability

The data presented in this study are available on request from the corresponding author.
